# Sex differences in the association between salivary telomere length and multimorbidity within the US Health & Retirement Study

**DOI:** 10.1093/ageing/afz071

**Published:** 2019-06-05

**Authors:** Claire L Niedzwiedz, Srinivasa Vittal Katikireddi, Jill P Pell, Daniel J Smith

**Affiliations:** 1 Institute of Health & Wellbeing, University of Glasgow, 1 Lilybank Gardens, Glasgow; 2 Medical Research Council/Chief Scientist Office Social and Public Health Sciences Unit, Institute of Health & Wellbeing, University of Glasgow

**Keywords:** Biomarkers, Epidemiology, Multimorbidities, Risk Factors, Telomeres, Older people

## Abstract

**Background:**

Telomere length is associated with several physical and mental health conditions, but whether it is a marker of multimorbidity is unclear. We investigated associations between telomere length and multimorbidity by sex.

**Methods:**

Data from adults (*N* = 5,495) aged ≥50 years were taken from the US Health and Retirement Study (2008–14). Telomere length was measured in 2008 from salivary samples. The cross-sectional associations between telomere length and eight chronic health conditions were explored using logistic regression, adjusting for confounders and stratified by sex. Logistic, ordinal and multinomial regression models were calculated to explore relationships between telomere length and multimorbidity (using a binary variable and a sum of the number of health conditions) and the type of multimorbidity (no multimorbidity, physical multimorbidity, or multimorbidity including psychiatric problems). Using multilevel logistic regression, prospective relationships between telomere length and incident multimorbidity were also explored.

**Results:**

In cross-sectional analyses, longer telomeres were associated with reduced likelihood of lung disease and psychiatric problems among men, but not women. Longer telomeres were associated with lower risk of multimorbidity that included psychiatric problems among men (OR=0.521, 95% CI: 0.284 to 0.957), but not women (OR=1.188, 95% CI: 0.771 to 1.831). Prospective analyses suggested little association between telomere length and the onset of multimorbidity in men (OR=1.378, 95% CI: 0.931 to 2.038) nor women (OR=1.224, 95% CI: 0.825 to 1.815).

**Conclusions:**

Although telomere length does not appear to be a biomarker of overall multimorbidity, further exploration of the relationships is merited particularly for multimorbidity including psychiatric conditions among men.

## Key points

- Few studies have investigated whether telomere length is a biomarker of multimorbidity
- Telomere length was not related to overall multimorbidity
- Among men, longer telomeres were associated with reduced risk of multimorbidity that included psychiatric problems

## Introduction

Multimorbidity (the coexistence of two or more chronic diseases) is an increasing global public health concern [1]. The prevalence of multimorbidity increases with age and affects over half of the older population [2]. It is associated with heightened mortality risk, decreased physical functioning and quality of life, and higher levels of inpatient and ambulatory healthcare [2, 3]. Multimorbidity is influenced by a number of social and lifestyle factors [4]; for example, women and individuals experiencing a disadvantaged socioeconomic position display an increased prevalence [2, 3]. However, to date, few studies have examined potential biological factors associated with multimorbidity. Improved understanding of the biological mechanisms that lead to the development of chronic diseases and multimorbidity is key to the development of new personalised treatments, prevention, and the reduction of escalating healthcare costs [5].

Telomere length has been widely proposed as a potential marker of biological ageing [6–8]. Telomeres are repetitive nucleotide sequences (of TTAGGG) at the end of chromosomes that protect the genome from damage and shorten with each cell division. Meta-analyses demonstrate that shorter telomeres are associated with a range of health outcomes, including cardiovascular disease [9], psychiatric disorders [10], and cancer [11]. However, the majority of included studies are limited by their cross-sectional design and few studies have examined telomere length in relation to multimorbidity [12].

It is plausible that telomere length could be a biomarker for multimorbidity and that shorter telomeres increase the risk of developing multimorbidity, with longer telomeres being potentially protective. A key factor that may influence this potential relationship is sex. Telomeres tend to be longer amongst women compared to men, with little evidence for any difference by age group [13]. This relationship is thought to be related to the actions of oestrogen and oxidative stress [13]. Oestrogen stimulates the production of telomerase, which is thought to protect against reactive oxygen species damage [14]. As multimorbidity tends to be more common in females, it is possible that any association between multimorbidity and telomere length may be modified by sex.

In this study we explore the relationships between telomere length and multimorbidity in cross-sectional and prospective analyses, using data from the US Health and Retirement Study and investigate potential differences by sex. We have three main objectives: (1) Examine the individual cross-sectional associations between telomere length and a number of chronic diseases; (2) Explore whether telomere length is related to multimorbidity in cross-sectional analyses, using different operationalisations of multimorbidity; (3) Prospectively investigate whether telomere length predicts the development of incident multimorbidity.

## Method

### Data

The Health and Retirement Study (HRS) is a nationally representative longitudinal survey of individuals aged over age 50 years in the USA. It began in 1992 and has continued to collect data every two years on the health and economic circumstances associated with ageing [15]. In 2008 (wave 9) the telomere lengths of 5,808 respondents who provided consent for a saliva sample were measured [16]. The HRS 2008 Telomere dataset is sponsored by the National Institute on Aging (grant number NIA U01AG009740) and the study was conducted by the University of Michigan [17]. The 2008 telomere data were linked to the RAND HRS dataset [18], which combines data for the 2008, 2010, 2012 and 2014 HRS waves. We included individuals aged 50 years and over (to represent older adults and those in early old age) during the 2008 wave of HRS who provided a saliva sample (*N* = 5,495), excluding individuals aged under 50 years (*N* = 119) and who were ineligible due to other reasons (e.g. living outside the U.S (*N* = 194)).

### Definition of multimorbidity

Participants were asked whether they have ever been told by a doctor that they had a health condition. The eight conditions assessed were: (1) high blood pressure; (2) diabetes or high blood sugar; (3) cancer or a malignant tumour (excluding minor skin cancer); (4) chronic lung disease (such as chronic bronchitis or emphysema); (5) a heart attack, coronary heart disease, angina, congestive heart failure, or other heart problems; (6) stroke; (7) emotional, nervous, or psychiatric problems; and (8) arthritis or rheumatism. A binary variable distinguishing those with no multimorbidity (none or one health condition) from those with multimorbidity (two or more health conditions) was generated and a multimorbidity score was created by summing the number of conditions present. This was top-coded at six due to the small number of individuals with seven or eight conditions when stratified by sex. We also categorised participants into groups depending on the type of multimorbidity: no multimorbidity (none or one health condition); physical multimorbidity (two or more physical health conditions); or multimorbidity including psychiatric problems (psychiatric problems and one or more physical health conditions).

### Telomere length

Telomere length data was obtained from the HRS 2008 Telomere dataset, which has been analysed previously in a number of studies [19, 20]. The telomeres were assayed using quantitative Polymerase Chain Reaction (qPCR) [21], which compared the telomere sequence copy number in each individual’s sample (T) to a single-copy gene copy number (S), producing a T/S ratio which is proportional to telomere length. The assays were conducted by Telome Health (Telomere Diagnostics, http://www.telomehealth.com/). Due to the positive skew we log transformed (using natural logarithm) the telomere length variable.

### Covariates

Age (years), ethnicity (non-Hispanic white, non-Hispanic Black, Hispanic, Other), education level (less than high school, high school or General Education Diploma (GED), some college, college or above), smoking (never smoked, previous smoker, current smoker) and body mass index (as a continuous variable) were included as covariates. A GED demonstrates a level of knowledge equivalent to a high school graduate. We investigated sex (male or female) as a potential moderating variable as we hypothesised the associations may be different between men and women. All statistical models also controlled for the plate number used to assay telomere length based on the different dilution factors used [22].

### Statistical analyses

Descriptive statistics for each variable were first examined using the cross-sectional data from 2008. The analyses followed a number of steps in line with our objectives described in the introduction. (1) We examined the cross-sectional age-adjusted relationships between telomere length (logged) and each of the eight health conditions in a series of logistic regression models and then adjusted for the additional covariates. These models were stratified by sex as interaction terms between telomere length and sex were statistically significant (*P* < 0.05) and to examine potential differences between men and women. (2) We then calculated logistic regression models predicting multimorbidity using a binary variable distinguishing those with and without multimorbidity (two or more health conditions versus none or one health condition). Using ordinal regression we then examined the association between telomere length (logged) and the sum of the number of health conditions present. Then, using multinomial regression we predicted the type of multimorbidity (no multimorbidity, physical multimorbidity, or multimorbidity including psychiatric problems) according to logged telomere length. For each operationalisation of multimorbidity we first calculated age-adjusted models, followed by models adjusting for the additional covariates. (3) We then investigated the prospective association between telomere length in 2008 and the development of multimorbidity during 2010 to 2014 (the following three waves) amongst individuals who were not multimorbid at baseline in 2008. The data were converted to long format and multilevel logistic regression models were calculated using logged telomere length as the explanatory variable and the development of multimorbidity (none or one health condition versus two or more health conditions) as the binary outcome variable. The models were stratified by sex and included waves nested within individuals, calculated using Stata’s xtlogit command. Due to the small sample size and lack of power we were unable to explore telomere length in relation to the onset of multimorbidity including psychiatric problems.

Sensitivity analyses were conducted excluding the participants (*N* = 270) with a telomere T/S ratio above 2.0, as these high values may be due to artefact in saliva samples [20]. Survey weights were used in the analyses to make the survey representative of the community-based population and the standard errors were adjusted for household clustering. A complete case analysis was performed excluding missing data for the covariates. For all cross-sectional analyses we included the same number of individuals in each model (2,272 men and 3,083 women). Analyses were performed using Stata/MP 15.1.

## Results

### Sample description

A total of 5,355 individuals (excluding those with missing covariate data, *N* = 140) were included in the cross-sectional sample, which was 55.2% female (Table 1). The mean age of participants was 66.8 years for men and 67.6 years for women, ranging from 50 to 100 years. Only 15.5% of men and 13.2% of women reported no health conditions at baseline. 23.8% of men and 24.6% of women had one condition, with 60.7% and 62.1% reporting multimorbidity (two or more health conditions). 48.9% of men and 44.4% of women had physical multimorbidity, and 11.8% of men and 17.7% of women had multimorbidity with psychiatric problems.

**Table 1.** Descriptive statistics for the sample (weighted %s)

	Male		Female	
	Mean	SD	Mean	SD
**Age**	66.8	9.4	67.6	9.9
**Body mass index**	28.4	5.2	28.2	6.5
**Telomere length**^a^	1.29	0.37	1.32	0.36
**Education level**	***N***	**%**	***N***	**%**
< High School	459	16.4	667	17.9
High school or General Education Diploma	706	29.0	1,188	38.0
Some college	489	24.3	706	24.6
College and above	618	30.3	522	19.5
**Ethnicity**				
Non-Hispanic white	1,753	81.3	2,272	79.7
Non-Hispanic Black	266	8.1	451	9.9
Hispanic	206	8.0	296	8.1
Other	47	2.6	64	2.3
**Smoking status**				
Never smoked	720	32.7	1,567	50.5
Previous smoker	1,251	52.2	1,139	36.2
Current smoker	301	15.1	377	13.3
**Health conditions (*N* and % with condition)**				
Psychiatric problems	246	12.9	561	19.1
High blood pressure	1,349	55.7	1,838	55.5
Arthritis	1,235	51.6	2,129	65.9
Diabetes	557	22.2	590	17.6
Stroke	242	8.8	224	6.7
Cancer	395	14.5	467	14.4
Lung disease	218	8.5	323	9.9
Heart problems	722	27.7	692	20.5
**No. of health conditions**				
0	285	15.5	322	13.2
1	496	23.8	722	24.6
2	606	26.4	832	25.9
3	492	19.7	678	20.3
4	239	8.9	325	9.6
5	106	3.8	134	4.2
6+	48	1.9	70	2.0
**Multimorbidity**				
No (none or 1 health condition)	781	39.3	1,044	37.9
Yes (2 or more health conditions)	1,491	60.7	2,039	62.1
**Multimorbidity type**				
None (none or 1 health condition)	781	39.3	1,044	37.9
Physical multimorbidity (2 or more physical health conditions)	1,259	48.9	1,512	44.4
Multimorbidity including psychiatric problems (2 or more health conditions that include psychiatric problems)	232	11.8	527	17.7
**Total**	**2,272**	**44.8**	**3,083**	**55.2**

^a^Geometric mean and interquartile range. *N*=number of individuals; SD=standard deviation.

### Cross-sectional relationship between telomere length and chronic health conditions

In age-adjusted models, longer telomeres were associated with reduced likelihood of lung disease for both men (OR=0.494, 95% CI: 0.277 to 0.883) and women (OR=0.522, 95% CI: 0.321 to 0.849) (Table 2, full results in Supplementary Tables S1 and S2). Longer telomeres were also related to the decreased likelihood of psychiatric problems among men only (OR=0.535, 95% CI: 0.303 to 0.945). Among men, once the models were adjusted for the additional covariates, the associations between telomere length, lung disease and psychiatric problems persisted. However, among women, adjustment for the covariates attenuated the association between telomere length and lung disease such that it was no longer statistically significant (Table 2). Among both genders, longer telomeres were also related to reduced likelihood of arthritis, cancer and heart problems, but these associations were not statistically significant.

**Table 2.** Associations between telomere length (logged) and health outcomes among 2,272 men and 3,083 women in the Health and Retirement Study

	Psychiatric problems	High blood pressure	Arthritis	Diabetes	Stroke	Cancer	Lung disease	Heart problems	Multimorbidity (no vs. yes)^a^	Multimorbidity (no. of health conditions)	Physical multimorbidity^a^	Multimorbidity including psychiatric problems^a^
	Logistic regression models									Ordinal regression model	Multinomial regression model	
	OR	OR	OR	OR	OR	OR	OR	OR	OR	OR	RRR	RRR
	[95% CI]	[95% CI]	[95% CI]	[95% CI]	[95% CI]	[95% CI]	[95% CI]	[95% CI]	[95% CI]	[95% CI]	[95% CI]	[95% CI]
**Age adjusted**												
**Men**	0.535^*^	1.144	0.898	1.258	0.936	0.739	0.494^*^	0.766	0.884	0.872	1.016	0.521^*^
Telomere length (logged)	[0.303,0.945]	[0.824,1.587]	[0.646,1.249]	[0.800,1.978]	[0.545,1.605]	[0.480,1.136]	[0.277,0.883]	[0.535,1.096]	[0.628,1.246]	[0.661,1.151]	[0.703,1.468]	[0.284,0.957]
**Women**	1.178	1.160	0.982	1.366	1.457	0.692	0.522^**^	0.877	0.972	1.004	0.886	1.188
Telomere length (logged)	[0.802,1.730]	[0.867,1.552]	[0.732,1.317]	[0.955,1.955]	[0.929,2.286]	[0.479,1.001]	[0.321,0.849]	[0.613,1.254]	[0.721,1.311]	[0.778,1.296]	[0.647,1.213]	[0.771,1.831]
**Adjusted for additional covariates**^1^												
**Men**	0.521^*^	1.061	0.863	1.056	0.890	0.701	0.529^*^	0.760	0.850	0.774	0.975	0.496^*^
Telomere length (logged)	[0.291,0.933]	[0.756,1.490]	[0.621,1.199]	[0.656,1.701]	[0.529,1.496]	[0.462,1.064]	[0.311,0.900]	[0.531,1.089]	[0.597,1.209]	[0.585,1.023]	[0.669,1.421]	[0.268,0.919]
**Women**	1.344	1.044	0.964	1.156	1.351	0.690	0.681	0.888	0.922	0.967	0.806	1.247
Telomere length (logged)	[0.924,1.955]	[0.756,1.442]	[0.707,1.315]	[0.807,1.656]	[0.895,2.040]	[0.465,1.022]	[0.434,1.069]	[0.633,1.245]	[0.664,1.279]	[0.749,1.248]	[0.572,1.135]	[0.801,1.942]

^1^Adjusted for age (years), ethnicity, education level, smoking status, Body Mass Index and plate number.

^a^No multimorbidity (none or one health condition) is the referent group.

CI=confidence interval; OR=odds ratio; RR=relative risk ratio. ^*^p < 0.05, ^**^p < 0.01, ^***^p < 0.001.

### Cross-sectional relationship between telomere length and multimorbidity

In the cross-sectional logistic regression analyses, telomere length was related to reduced likelihood of multimorbidity in men (OR=0.884, 95% CI: 0.628 to 1.246) and women (OR=0.972, 95% CI: 0.721 to 1.311), but these associations were not statistically significant when using the binary multimorbidity variable as the outcome (Table 2). Similar results were found for men when using the number of conditions as the outcome and conducting ordinal regression. When examining the association between telomere length and the type of multimorbidity, we find that among men, longer telomeres were related to lower risk of multimorbidity including psychiatric problems, compared to no multimorbidity (RRR = 0.521, 95% CI: 0.284 to 0.957). This association persisted when adjusting for the covariates. The association also remained after changing the baseline referent category to physical multimorbidity. There was little association found between telomere length and the type of multimorbidity among women. Sensitivity analysis excluding participants with a T/S ratio of above 2 did not affect the substantive results.

### Prospective association between telomere length and multimorbidity

A total of 1,989 individuals (59.0% women) had no multimorbidity at baseline in wave 9. By wave 12, 602 individuals had developed multimorbidity. In age-adjusted multilevel logistic regression models (Table 3), telomere length was not associated with the development of multimorbidity (defined as two or more health conditions) for either sex and the direction of the association was in the opposite to that expected if shorter telomeres predispose the development of multimorbidity. Among men, only increased age was associated with the development of multimorbidity (OR=1.035, 95% CI: 1.021 to 1.050). Both increased age (OR=1.035, 95% CI: 1.023 to 1.047) and BMI (OR=1.036, 95% CI: 1.015 to 1.057) were related to the onset of multimorbidity among women.

**Table 3.** Multilevel logistic regression model results predicting the development of multimorbidity (2 or more health conditions, referent category is none or one health condition)

	Men		Women	
	Model 1	Model 2	Model 1	Model 2
	OR	OR	OR	OR
	[95% CI]	[95% CI]	[95% CI]	[95% CI]
**Telomere length** (logged)	1.378	1.276	1.224	1.279
	[0.931,2.038]	[0.858,1.897]	[0.825,1.815]	[0.865,1.892]
**Age** (years)	1.034^***^	1.035^***^	1.027^***^	1.035^***^
	[1.021,1.048]	[1.021,1.050]	[1.016,1.038]	[1.023,1.047]
**Education Level** (ref = < high school)				
High school or General Education Diploma		0.939		1.058
		[0.630,1.400]		[0.751,1.493]
Some college		0.833		0.895
		[0.535,1.297]		[0.615,1.303]
College & above		0.911		1.192
		[0.601,1.379]		[0.807,1.761]
**Body Mass Index**		1.015		1.036^***^
		[0.987,1.045]		[1.015,1.057]
**Smoking status** (ref=never smoked)				
Previous smoker		1.020		0.889
		[0.772,1.349]		[0.695,1.136]
Current smoker		1.066		1.272
		[0.713,1.594]		[0.909,1.780]
**Ethnicity** (ref=Non-Hispanic white)				
Non-Hispanic Black		0.937		1.251
		[0.616,1.425]		[0.895,1.749]
Hispanic		1.222		1.295
		[0.797,1.873]		[0.892,1.881]
Other		0.681		1.091
		[0.323,1.435]		[0.513,2.319]

CI=confidence interval; OR=odds ratio. ^*^p < 0.05, ^**^p < 0.01, ^***^p < 0.001.

Model 1: Adjusted for age; Model 2: Adjusted for age, ethnicity, education level, smoking status, Body Mass Index and plate number.

## Discussion

We hypothesised that telomere length could act as a biomarker of multimorbidity. However, there was little overall evidence to support this hypothesis. Among both men and women, longer telomeres related to the reduced likelihood of lung disease, but this association was attenuated among women after adjustment for potential confounding factors. For men, longer telomeres were also associated with the decreased likelihood of psychiatric problems, an association that persisted following adjustment for covariates. No evidence was found for an association between telomere length and multimorbidity among women. Findings were similar for men apart from when distinguishing between the types of multimorbidity. Longer telomeres were related to the reduced risk of multimorbidity including psychiatric problems, compared to no multimorbidity (which may have included psychiatric problems as a singular condition), and this association was not observed for men with physical multimorbidity. In the prospective analyses, telomere length was not related to the onset of multimorbidity for men or women. Although this suggests that telomere length is not predictive of the onset of multimorbidity, this may be due to a lack of statistical power. Further prospective research with larger sample sizes and repeated telomere length measurements to record change, as well as meta-analyses and studies that assess causality (e.g. using a Mendelian Randomisation approach) are required to fully understand the relationships between telomere length, psychiatric problems and comorbid physical conditions.

We observed that longer telomeres were related to reduced likelihood of lung disease and psychiatric problems among men in cross-sectional analyses. This is consistent with previous research demonstrating that shorter telomeres tend to be found amongst people with psychiatric illness, particularly depressive disorders [10]. Previous studies have also demonstrated a modest association between shorter telomeres, lung function and chronic obstructive pulmonary disease [19, 23]. The sex differences noted in our study have been observed in other studies examining the relationship between telomere length and psychiatric disorders. For example, in a prospective study investigating depression, generalised anxiety disorder, post-traumatic stress disorder and telomere erosion, Shalev *et al.* found associations among men but not women [24]. They suggest that men may be more prone than women to physiological changes related to psychiatric disorders, which are also implicated in telomere function [24], such as the hypothalamic-pituitary-adrenal axis response to stress [25], elevated proinflammatory cytokines [26], and greater oxidative stress markers [27]. More recent evidence from Mendelian Randomisation studies suggest a more complicated picture. Longer telomeres were associated with the increased risk of several types of cancer, shorter telomeres related to increased risk of cardiovascular disease and interstitial lung disease, but little evidence was found for psychiatric disorders [28], suggesting observational studies are likely to be affected by confounding and reverse causation.

### Strengths and limitations

An important strength of our study is the relatively large sample size allowing stratification by sex. Our study was also multi-ethnic and included both cross-sectional and prospective analyses. It is possible that relationships could differ by ethnicity, which we have not explored in this study. Whilst numerous studies have investigated the association between single diseases and telomere length, few have examined multimorbidity specifically. However, our study is limited by the self-reported nature of the phenotype data, which may be subject to recall bias. As participants were not asked about every physical or mental health condition that they had experienced, it is possible that some respondents had other conditions that were not recorded. The questionnaire item relating to psychiatric problems was also broad and did not assess the severity of condition, or whether participants had experienced multiple mental health conditions. This was also an issue for some of the other disease categories, such as cancer, with previous research demonstrating that different types of cancer may have variable relationships with telomere length [28]. Due to the high proportion of individuals with multimorbidity at baseline, our prospective analyses also lacked power. In addition, the results may not be generalisable to those aged under 50 years. Telomeres were also only measured at one time point and it is possible that the cross-sectional relationships found in this study may be subject to reverse causality and confounding. Future studies could investigate the direction of causality using a Mendelian Randomisation approach [28]. Our study also only analysed telomere length derived from saliva samples. It is possible that different results may be obtained from other tissue types (e.g. venous blood) as different cell types have varying rates of division and therefore different rates of telomere attrition [29]. Few previous studies have compared telomere lengths obtained from blood and saliva [30], but those that have find they are correlated, with telomeres derived from salivary DNA tending to be longer than those derived from blood samples [31, 32].

## Conclusion

We examined the cross-sectional and prospective relationships between telomere length and multimorbidity in a cohort of older adults. In cross-sectional analyses, longer telomeres were associated with reduced risk of multimorbidity that included psychiatric problems, among men only. Prospective analyses did not find telomere length to be predictive of the development of multimorbidity, but our analyses lacked statistical power. Further prospective studies with larger samples and studies that assess the direction of causality (e.g. via Mendelian Randomisation) are needed to investigate the relationship between telomere length and specific types of multimorbidity, such as psychiatric disorders and cardiovascular disease.

## Supplementary Material

Supplementary Data

## Notes

**Acknowledgements:** The HRS *2008 Telomere* data set is sponsored by the National Institute on Aging (grant number NIA U01AG009740) and was conducted by the University of Michigan [17].

**Declaration of Conflict of interest:** None.

**Declaration of Sources of Funding:** This work was supported by the Medical Research Council (Skills Development Fellowship grant number MR/R024774/1 to CLN), National Health Service (NHS) Research Scotland Senior Clinical Fellowship (grant number SCAF/15/02 to SVK), the Medical Research Council (grant numbers MC_UU_12017/13, MC_UU_12017/15 to SVK) and the Chief Scientist Office (grant numbers SPHSU13, SPHSU15 to SVK), the Medical Research Council (Mental Health Data Pathfinder Award grant number MC_PC_17217 to DJS). The funders had no role in the study design; collection, analysis and interpretation of data; the writing of the articles; and in the decision to submit it for publication.
