# Supplementary material for: Sex differences in the association between salivary telomere length and multimorbidity within the US Health & Retirement Study
Source: Age Ageing. 2019 Jun 5;48(5):703–10. doi: 10.1093/ageing/afz071 (PMC6984958; doi:10.1093/ageing/afz071)
Supplement: Supplementary Data [file afz071_aa-19-0032-file003.docx]

**Online Supplementary Data**

Supplementary Table S1: Associations between telomere length and health outcomes among 2272 men in the Health and Retirement Study adjusted for covariates

|  | **Psychiatric problems** | **High blood pressure** | **Arthritis** | **Diabetes** | **Stroke** | **Cancer** | **Lung disease** | **Heart problems** |
| --- | --- | --- | --- | --- | --- | --- | --- | --- |
|  | OR  [95% CI] | OR  [95% CI] | OR  [95% CI] | OR  [95% CI] | OR  [95% CI] | OR  [95% CI] | OR  [95% CI] | OR  [95% CI] |
| **Telomere length** (logged) | 0.521^*^ [0.291,0.933] | 1.061 [0.756,1.490] | 0.863 [0.621,1.199] | 1.056 [0.656,1.701] | 0.890 [0.529,1.496] | 0.701 [0.462,1.064] | 0.529^*^ [0.311,0.900] | 0.760 [0.531,1.089] |
| **Age (years)** | 0.955^***^ [0.938,0.973] | 1.038^***^ [1.026,1.051] | 1.039^***^ [1.027,1.051] | 1.031^***^ [1.018,1.045] | 1.059^***^ [1.041,1.077] | 1.067^***^ [1.051,1.082] | 1.033^***^ [1.015,1.053] | 1.063^***^ [1.050,1.076] |
| **Ethnicity** (ref=Non-Hispanic white) |  |  |  |  |  |  |  |  |
| Non-Hispanic Black | 0.887 [0.537,1.465] | 2.550^***^ [1.777,3.660] | 0.697^*^ [0.501,0.968] | 1.540^*^ [1.054,2.250] | 1.600 [0.946,2.707] | 1.143 [0.769,1.700] | 0.628 [0.346,1.141] | 0.561^**^ [0.378,0.834] |
| Hispanic | 0.573 [0.293,1.120] | 1.078 [0.738,1.576] | 0.666^*^ [0.449,0.989] | 1.697^*^ [1.095,2.630] | 0.729 [0.341,1.556] | 0.181^***^ [0.089,0.369] | 0.325^*^ [0.111,0.955] | 0.638^*^ [0.418,0.972] |
| Other | 0.519 [0.149,1.811] | 0.491 [0.215,1.125] | 0.461^*^ [0.223,0.954] | 2.702^*^ [1.199,6.092] | 1.842 [0.649,5.228] | 0.375^*^ [0.142,0.989] | 0.523 [0.138,1.989] | 0.771 [0.329,1.809] |
| **Education Level** (ref= < high school) |  |  |  |  |  |  |  |  |
| High school or General Education Diploma | 0.702 [0.434,1.134] | 1.306 [0.946,1.803] | 0.803 [0.585,1.103] | 0.742 [0.517,1.066] | 1.387 [0.827,2.327] | 0.984 [0.671,1.442] | 0.826 [0.523,1.305] | 0.977 [0.709,1.345] |
| Some college | 0.716 [0.437,1.175] | 1.166 [0.823,1.651] | 0.834 [0.592,1.175] | 0.820 [0.556,1.210] | 1.184 [0.649,2.160] | 0.840 [0.548,1.287] | 0.625 [0.364,1.071] | 0.891 [0.626,1.268] |
| College & above | 0.733 [0.443,1.213] | 0.979 [0.696,1.379] | 0.503^***^ [0.360,0.703] | 0.497^***^ [0.331,0.745] | 1.046 [0.590,1.852] | 0.866 [0.567,1.321] | 0.347^***^ [0.195,0.619] | 0.627^**^ [0.441,0.891] |
| **Smoking status** (ref=never smoked) |  |  |  |  |  |  |  |  |
| Previous smoker | 1.354 [0.911,2.012] | 1.388^**^ [1.096,1.757] | 1.312^*^ [1.037,1.660] | 0.969 [0.736,1.276] | 1.103 [0.768,1.584] | 0.977 [0.718,1.329] | 3.922^***^ [2.167,7.100] | 1.173 [0.917,1.500] |
| Current smoker | 1.704^*^ [1.026,2.829] | 1.320 [0.931,1.872] | 1.059 [0.753,1.490] | 0.680 [0.425,1.088] | 2.164^**^ [1.289,3.632] | 0.866 [0.521,1.442] | 6.391^***^ [3.193,12.789] | 1.497^*^ [1.006,2.228] |
| **Body Mass Index** | 1.029 [0.996,1.063] | 1.100^***^ [1.070,1.132] | 1.059^***^ [1.035,1.083] | 1.105^***^ [1.077,1.133] | 1.019 [0.978,1.061] | 1.029 [0.999,1.059] | 1.027 [0.979,1.076] | 1.044^***^ [1.020,1.068] |

*Models also adjusted for plate number. CI=confidence interval; OR=odds ratio; ref=reference category. ^*^ p < 0.05, ^**^ p < 0.01, ^***^ p < 0.001*

Supplementary Table S2: Associations between telomere length and health outcomes among 3083 women in the Health and Retirement Study adjusted for covariates

|  | **Psychiatric problems** | **High blood pressure** | **Arthritis** | **Diabetes** | **Stroke** | **Cancer** | **Lung disease** | **Heart problems** |
| --- | --- | --- | --- | --- | --- | --- | --- | --- |
|  | OR  [95% CI] | OR  [95% CI] | OR  [95% CI] | OR  [95% CI] | OR  [95% CI] | OR  [95% CI] | OR  [95% CI] | OR  [95% CI] |
| **Telomere length** (logged) | 1.344 [0.924,1.955] | 1.044 [0.756,1.442] | 0.964 [0.707,1.315] | 1.156 [0.807,1.656] | 1.351 [0.895,2.040] | 0.690 [0.465,1.022] | 0.681 [0.434,1.069] | 0.888 [0.633,1.245] |
| **Age** (years) | 0.984^**^ [0.972,0.996] | 1.055^***^ [1.044,1.065] | 1.055^***^ [1.043,1.066] | 1.020^**^ [1.007,1.033] | 1.070^***^ [1.053,1.088] | 1.034^***^ [1.021,1.046] | 1.034^***^ [1.018,1.050] | 1.050^***^ [1.038,1.062] |
| **Ethnicity** (ref=Non-Hispanic white) |  |  |  |  |  |  |  |  |
| Non-Hispanic Black | 0.514^***^ [0.359,0.734] | 2.140^***^ [1.568,2.921] | 0.927 [0.691,1.244] | 2.198^***^ [1.624,2.976] | 1.790^*^ [1.119,2.865] | 0.685 [0.461,1.018] | 0.676 [0.434,1.052] | 1.223 [0.912,1.640] |
| Hispanic | 0.856 [0.578,1.267] | 0.860 [0.619,1.195] | 0.622^**^ [0.444,0.872] | 1.400 [0.970,2.022] | 0.425 [0.171,1.056] | 1.106 [0.700,1.748] | 0.285^**^ [0.128,0.632] | 0.468^***^ [0.308,0.710] |
| Other | 1.696 [0.869,3.312] | 1.412 [0.721,2.765] | 1.495 [0.720,3.107] | 3.423^***^ [1.773,6.608] | 1.718 [0.589,5.008] | 0.327^*^ [0.114,0.935] | 0.794 [0.323,1.955] | 0.681 [0.321,1.446] |
| **Education Level** (ref= < high school) |  |  |  |  |  |  |  |  |
| High school or General Education Diploma | 0.655^**^ [0.486,0.882] | 0.675^**^ [0.514,0.886] | 0.774 [0.589,1.017] | 0.651^**^ [0.482,0.879] | 0.828 [0.565,1.211] | 1.425^*^ [1.019,1.991] | 0.577^**^ [0.402,0.828] | 0.601^***^ [0.459,0.786] |
| Some college | 0.499^***^ [0.352,0.708] | 0.555^***^ [0.411,0.749] | 0.685^*^ [0.510,0.920] | 0.521^***^ [0.369,0.737] | 0.608 [0.360,1.027] | 1.475^*^ [1.022,2.130] | 0.508^**^ [0.332,0.777] | 0.541^***^ [0.397,0.737] |
| College & above | 0.560^**^ [0.384,0.818] | 0.481^***^ [0.349,0.664] | 0.631^**^ [0.457,0.872] | 0.408^***^ [0.268,0.623] | 0.595 [0.344,1.031] | 1.534^*^ [1.020,2.308] | 0.374^***^ [0.225,0.622] | 0.350^***^ [0.242,0.508] |
| **Smoking status** (ref=never smoked) |  |  |  |  |  |  |  |  |
| Previous smoker | 1.484^**^ [1.167,1.888] | 0.975 [0.804,1.183] | 1.152 [0.938,1.415] | 0.996 [0.776,1.279] | 1.360 [0.966,1.915] | 1.380^**^ [1.080,1.764] | 3.444^***^ [2.465,4.811] | 1.281^*^ [1.027,1.596] |
| Current smoker | 2.102^***^ [1.517,2.913] | 1.046 [0.781,1.400] | 1.223 [0.911,1.642] | 1.303 [0.915,1.857] | 1.285 [0.701,2.353] | 0.701 [0.456,1.078] | 9.447^***^ [6.313,14.137] | 1.616^**^ [1.154,2.263] |
| **Body Mass Index** | 1.032^***^ [1.013,1.051] | 1.097^***^ [1.078,1.116] | 1.081^***^ [1.062,1.100] | 1.115^***^ [1.094,1.136] | 1.026 [0.988,1.065] | 1.023^*^ [1.001,1.045] | 1.057^***^ [1.033,1.082] | 1.030^***^ [1.013,1.047] |

*Models also adjusted for plate number. CI=confidence interval; OR=odds ratio; Ref=reference category. ^*^ p < 0.05, ^**^ p < 0.01, ^***^ p < 0.001*
